# Osteoprotective Effect of *Radix Scutellariae* in Female Hindlimb-Suspended Sprague-Dawley Rats and the Osteogenic Differentiation Effect of Its Major Constituent

**DOI:** 10.3390/molecules22071044

**Published:** 2017-07-03

**Authors:** Guangwei Zhang, Chenrui Li, Yinbo Niu, Qi Yu, Yulong Chen, Enqi Liu

**Affiliations:** 1 Research Institute of Atherosclerotic Disease, College of Clinical Medicine, Xi’an Medical University, No.1Xinwang Road, Xi’an 710021, China; zgw_1979@163.com (G.Z.); qiyu6028@hotmail.com (Q.Y.); chenyunlong.0901@stu.xjtu.edu.cn (Y.C.); 2 Laboratory Animal Center, Xi′an Jiaotong University Health Science Center, Xi’an Jiaotong University, Xi’an 710061, China; 3 Key Laboratory for Space Biosciences & Biotechnology, School of Life Sciences, Northwestern Polytechnical University, 127 Youyixi Road, Xi’an 710072, China; lichenrui525@nwpu.edu.cn (C.L.); ybniuniu@nwpu.edu.cn (Y.N.)

**Keywords:** *Radix Scutellariae*, baicalin, disuse-induced osteoporosis, osteogenic differentiation, bone marrow derived mesenchymal stem cells

## Abstract

A number of medicinal herbs have demonstrated therapeutic effects for the prevention and treatment of disuse-induced osteoporosis. As a common ingredient in proprietary traditional Chinese medicines, the anti-osteoporosis effects of *Radix Scutellariae* extract (RSE, 50 mg/kg/day) were evaluated in a hindlimb suspended rat model. Bone mineral density (BMD) was measured by dual-energy X-ray absorptiometry, and the micro-architecture observed by MicroCT assay with bone biomechanical properties evaluated by a three-point bending test. To elucidate potential mechanisms, the osteogenic differentiation effect of baicalin as the most abundant ingredient in RSE was investigated in rat bone marrow derived mesenchymal stem cells (rBMSC). After drug administration for 42 days, tibia-BMD was significantly increased to 0.176 ± 0.007 and 0.183 ± 0.011 g/cm^2^ and f-BMD was enhanced to 0.200 ± 0.017 and 0.207 ± 0.021 g/cm^2^ for RSE and ALE treatment, respectively, whereas tibia-BMD and femur-BMD of the HLS group were 0.157 ± 0.009 and 0.176 ± 0.008 g/cm^2^. Deterioration of bone trabecula microstructure was improved by RSE and ALE with increased morphological parameters such as bone volume fraction, trabecular thickness, and trabecular number, as well as connectivity density compared to the HLS group (*p* < 0.01). A three-point bending test suggested that bone mechanical strength was also enhanced by RSE and ALE treatments with increased maximum stress, young’s modulus, maximum load, and stiffness compared to those of the HLS group (*p* < 0.05). Besides, serum TRACP levels were significantly suppressed by RSE and ALE treatments. Furthermore, in vitro studies demonstrated that baicalin significantly increased ALP activities and the formation of mineralized nodules in rBMSC. Conclusively, supplementation of RSE could significantly prevent weightlessness induced osteoporosis, which might attribute to the osteogenic differentiation enhancement effect of baicalin.

## 1. Introduction

Osteoporosis remains a global health issue, and several risk factors account for the occurrence of osteoporosis such as age, hormone fluctuation, chronic inflammatory diseases, medications, and mechanical stress [1,2,3]. Physical inactivity would cause disuse-induced osteoporosis, which not only threatens the safety and health of astronauts during space flight, but also increases the susceptibility to fractures in patients and elderly requiring prolonged bed rest [4]. A study showed that long-duration space flights caused a loss of bone mineral by 2–9% among 45 crew members [5]. After 17 weeks of horizontal bed rest, a 3.4% decrease of total hip bone mineral density (BMD) was observed in 18 volunteers [6]. The hindlimb suspension is frequently employed to simulate microgravity, but most of the studies were conducted in male rats [7]. With more female astronauts recruited in space flight, the impact of microgravity on females warrants an in-depth investigation.

For the treatment of osteoporosis, current commercial medications (including biphosphonates, parathyroid hormone, selective estrogen receptor, calcitonin, and hormone replacement therapy) are proven effective, but a number of adverse effects have been observed such as serious upper gastrointestinal bleeding for bisphosphonates and carcinogenicity after long-term intake of hormones [8,9]. As a result, efforts are made to seek new drug candidates from natural resources with high potency and low toxicity. A number of herbal medicines have exhibited significant-anti-osteoporotic effect to prevent osteoporosis induced by estrogen deficiency or physical inactivity, such as *Epimedium* L., *Radix Dipsaci*, *Eucommia ulmoides* Oliv. cortex, *Rhizoma Drynariae*as, as well as herbal formulation containing Epimedii Herba, Ligustri Lucidi Fructus, and Psoraleae Fructus [10].

*Radix Scutellariae* is a common medicinal herb which is extensively employed in traditional formulation and modern herbal preparations [11]. Flavonoids are the most abundant components in *Radix Scutellariae* extract (RSE), among which baicalin has been shown to induce osteoblastic differentiation via the Wnt/β-catenin signaling pathway and baicalein could inhibit osteoclast differentiation and induce mature osteoclast apoptosis [12,13]. Most importantly, our previous study demonstrated that RSE effectively inhibited the bone loss in male hindlimb suspended rats [14]. In the current study, female hindlimb suspended rats were used to evaluate the effect of RSE to increase the bone mineral density and improve the micro-architecture of trabecular bone. Gender differences of Sprague-Dawley rats in the occurrence of disuse-induced osteoporosis were further discussed. As the most abundant ingredient in RSE, the effect of baicalin on the osteogenic differentiation of rat bone marrow derived stem cells was also evaluated in vitro.

## 2. Results

### 2.1. Body Weights

**Figure 1:**
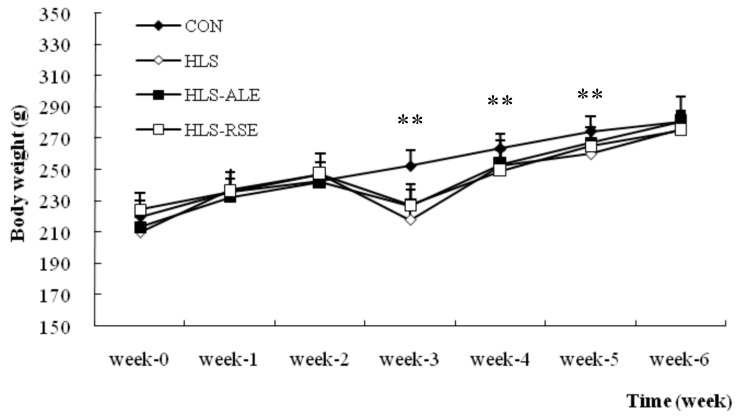
Measurements of rat body weights within various treatment groups. CON: control group; HLS: hindlimb suspended group; HLS-ALE: group administrated with alendronate (2 mg/kg/day); HLS-RSE: group administrated with *Radix Scutellariae* extract (50 mg/kg/day). Values are presented by means ± SD, *n* = 6, * versus CON group: ** *p* < 0.01.

The trends of body weight changes during the experiment were plotted and shown in Figure 1. During the first two weeks, body weights increased with time. On the third week, the body weights in HLS, HLS-ALE, and HLS-RSE were significantly decreased compared with CON group (*p* < 0.01). Then the body weight recovered with time and there was no significant difference in body weight in the various treatment groups at the end of experiment.

Compared with female rats, male rats showed a higher increase between final and initial body weight for CON (58.79% vs. 27.64%), HLS (60.54% vs. 31.10%), HLS-ALE (52.72% vs. 31.68%), and HLS-RSE (49.67% vs. 22.55%) groups [14].

### 2.2. BMD Evaluation of Femur and Tibia

**Figure 2:**
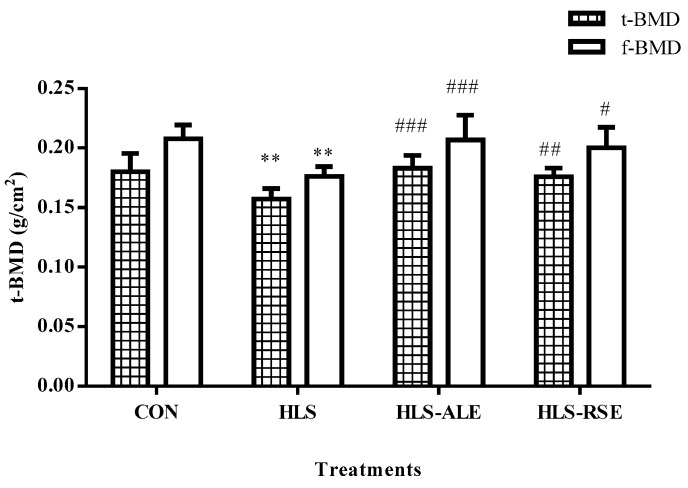
Bone mineral density of proximal tibia (t-BMD) and distal femur (f-BMD) within various treatment groups (g/cm^2^). CON: control group; HLS: hindlimb suspended group; HLS-ALE: group administrated with alendronate (2 mg/kg/day); HLS-RSE: group administrated with *Radix Scutellariae* extract (50 mg/kg/day). Values are presented by means ± SD, *n* = 6, * versus CON group: ** *p* < 0.01; ^#^ versus HLS group: ^#^ *p* < 0.05; ^##^ *p* < 0.01; ^###^ *p* < 0.001.

Impact of ALE and RSE treatment on proximal tibia and distal femur BMD were shown in Figure 2. Compared with CON rats, hindlimb suspension induced significant loss of BMD in both distal femur and proximal tibia (*p* < 0.001). Compared with the HLS group, ALE and RSE treatments significantly increased BMD values (*p* < 0.05) in the tibia and femur.

Based on our previous data in male rats [14], hindlimb suspension decreased t-BMD by 24.13% for male rats and 12.76% for female rats, respectively, while the f-BMD was reduced by 28.62% and 15.24% for male and female rats, suggesting the HLS treatment had more significant impact on BMD value for male rats.

### 2.3. Micro-Architecture of Femoral Trabecula

**Figure 3:**
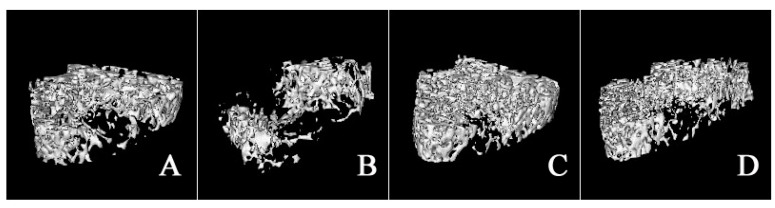
Representative 3-D images of bone trabeculain the distal femoral metaphysic region: (**A**) CON; (**B**) HLS; (**C**) HLS-ALE; (**D**) HLS-RSE (*n* = 6). CON: control group; HLS: hindlimb suspended group; HLS-ALE: group administrated with alendronate (2 mg/kg/day); HLS-RSE: group administrated with *Radix Scutellariae* extract (50 mg/kg/day).

**Figure 4:**
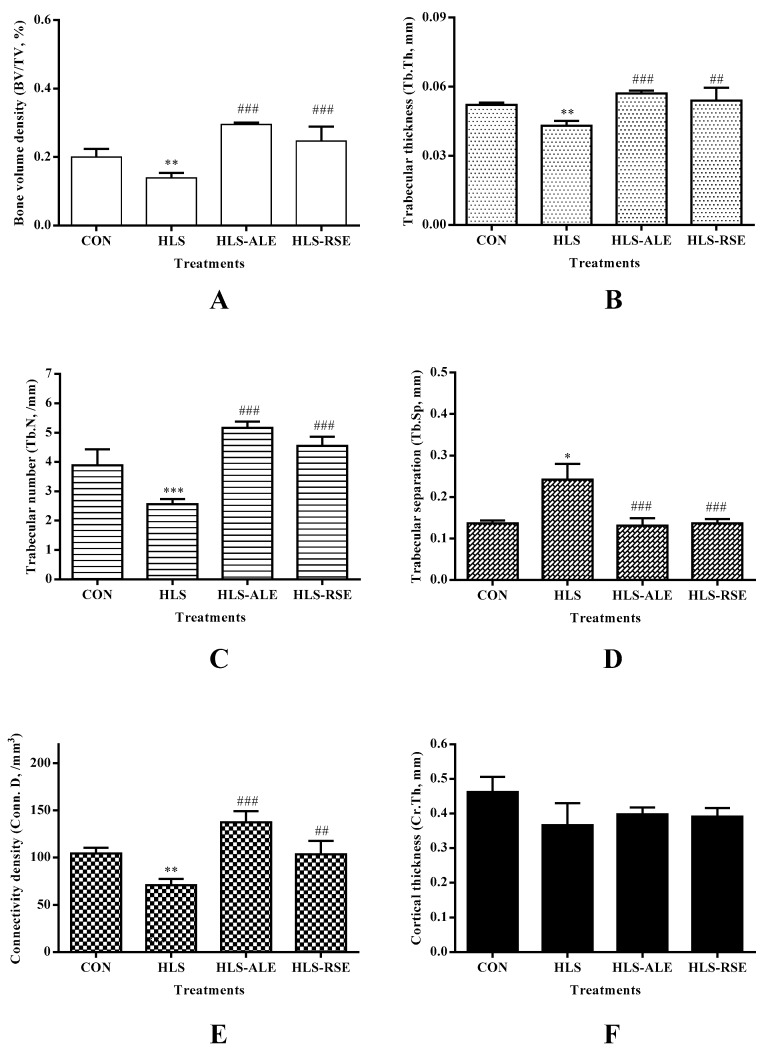
Measurements of femoral trabecula within various treatment groups: (**A**) bone volume density (BV/TV); (**B**) trabecular thickness (Tb.Th); (**C**) trabecular number (Tb.N); (**D**) trabecular separation (Tb.Sp), (**E**) connectivity density (Conn. D) as well as (**F**) cortical thickness (Cr. Th) (*n* = 6). CON: control group; HLS: hindlimb suspended group; HLS-ALE: group administrated with alendronate (2 mg/kg/day); HLS-RSE: group administrated with *Radix Scutellariae* extract (50 mg/kg/day); * versus CON group: * *p* < 0.05, ** *p* < 0.01, ***p < 0.001; ^#^ versus HLS group: ^##^ *p* < 0.01, ^###^ *p* < 0.001.

HLS generated a deteriorated condition of femoral trabecula according to the 3-D images or the corresponding architectural parameters of a MicroCT assay [15]. Representative 3-D images of trabecular bone within various treatment groups are shown in Figure 3. Micro-architecture properties of femoral trabecula were analyzed by the microCT Evaluation Program (V2.1.2, GE healthcare, Madison, WI, USA) and the results were shown in Figure 4. Compared with the CON group, hindlimb suspension could significantly decrease BV/TV (−30.70%), Tb.Th (−16.37%), Tb.N (−34.11%), and Conn. D (−32.20%), whereas Tb.Sp was increased by 76.51%. In addition, cortical thickness was not influenced by HLS or drug treatment.

By comparing the micro-architecture properties of trabecular femur between both genders, hindlimb suspension generated greater decrease in BV/TV (−77.60%), Tb.Th (−31.17%), Tb.N (−70.43%), and Conn. D (−99.13%) for male rats. The enhancement in Tb.Sp was also greater in male rats (295.04%). For both genders, ALE and RSE treatment significantly improved the impaired micro-architecture parameters induced by hindlimb suspension (*p* < 0.01) [14].

### 2.4. Bone Biomechanical Test

The influence of hindlimb suspension on biomechanical strength of femoral diaphysis was evaluated using a three-point bending test. As expected, bone structural biomechanical parameters (i.e., maximum load and stiffness), as well as bone material biomechanical parameters (i.e., maximum stress and Young’s modulus) were significantly reduced after hindlimb suspension (Table 1). Hindlimb unloading significantly reduced femoral bone strength, with the decreased bone structure indices including maximum stress (*p* < 0.05), Young’s modulus (*p* < 0.05), maximum load (*p* < 0.01), and stiffness (*p* < 0.001). Compared with the parameters in the HLS group, the administration of ALE and RSE could significantly reverse the decreased bone biomechanical strength.

Similar to the other indices, biomechanical parameters declined to a greater extent for male rats than for female rats including Young’s modulus (−38.76% vs. −23.81%), maximum load (−31.43% vs. −19.80%), and stiffness (−50.14% vs. −39.32%).

### 2.5. Assay of Bone Turnover Markers in Serum

Hindlimb suspension induced bone loss was coupled by accelerated bone remodeling which was evidence by increased bone turnover markers in serum. As shown in Table 2, the levels of Ca and P in various treatment groups were comparable to those in the CON group. Compared with the HLS group, the treatment of ALE and RSE could significantly suppress the HLS-induced elevation in the serum concentration of TRACP (*p* < 0.05), whereas the BAP level was not influenced by neither HLS, nor drug administrations.

In addition, HLS did not change the BAP level in female rats and the TRACP levels increased to a greater extent in male rats than in female rats (35.15% vs. 24.57%), indicating bone turnover accelerated much more in male rats than in female rats.

### 2.6. The Effect of Baicalin to Enhance Osteogenic Differentiation of rBMSC

**Figure 5:**
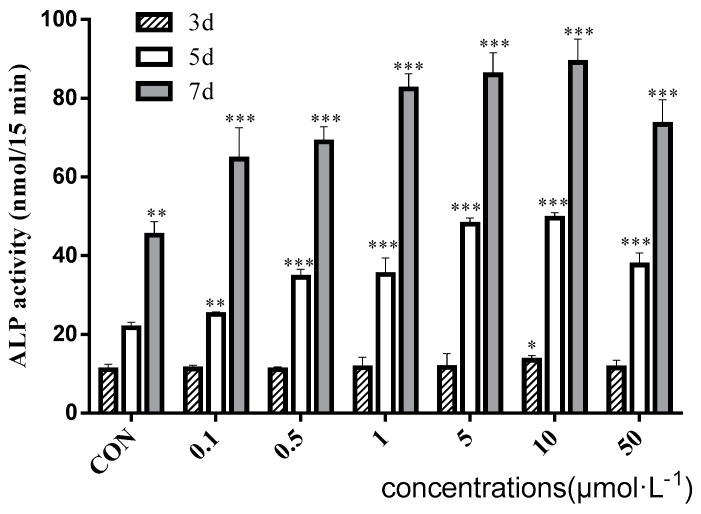
Effect of baicalin on ALP activity at various concentrations (*n* = 6). * *p* < 0.05, ** *p* < 0.01, *** *p* < 0.001.

**Figure 6:**
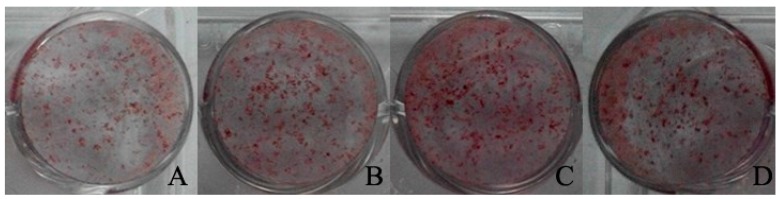
Effect of baicalin at various concentrations on mineralized nodule formation 12 days after osteogenic induction. (**A**) CON; (**B**) baicalin at 1 μM; (**C**) baicalin at 10 μM; (**D**) baicalin at 50 μM.

As shown in Figure 5, the ALP activity increased with time. Baicalin dose-dependently enhanced the ALP activity at day 5 and day 7. Baicalin at 10 μM showed a highest effect of osteogenic differentiation on rBMSC and this effect decreased when the concentration reached 50 μM. After osteogenic induction for 12 days, the mineralized nodules were formed in the presence and absence of baicalin (Figure 6). Compared with the CON group, baicalin at 1, 10, and 50μM significantly increased the area, number, and intensity of mineralized nodules based on the analysis via Image-Pro Plus 6.0 (Table 3). Baicalin at 10 μM demonstrated the highest effect to enhance the formation of mineralized nodules. All the results indicated that baicalin showed significant efficacy to enhance the osteogenic differentiation of rBMSC, which might increase bone formation and account for the anti-osteoporosis effect of RSE.

## 3. Discussion

Mechanical load is crucial for the maintenance of bone mass and strength, and physical inactivity would accelerate the bone microarchitecture deterioration and demineralization [16]. To determine the effect of weightlessness or mechanical unloading on skeletal system, a ground-based hindlimb suspended rats/mouse model has been established as an alternative to expensive experiments during space flight [17]. Based on previous publications, most of the studies used male rats/mice, and the impact of mechanical unloading on female rats has been rarely discussed. In the current study, female rats were employed as the modeled animal and their responses to hindlimb suspension were compared with those of male rats based on our previous study [14]. With regard to weightlessness or mechanical unloading induced osteoporosis, alendronate is usually chosen as the positive control. Alendronate is a kind of bisphosphonate, which is the most widely used medication in the treatment of skeletal disorders such as osteoporosis, due to its significant pharmacological effect of inhibition on bone resorption [18,19].

Although mechanical unloading generated bone loss in both genders, male rats were found to be more sensitive to hindlimb suspension than female rats. HLS induced a stronger effect on bodyweight changes in male rats than in female rats. In the work by Qi et al., a similar change in bodyweight was observed in both genders [20]. Age might contribute to the different results. In our study, younger rats (two-month-old) were employed, whereas Qi et al., used rats that were six-months-old. The impact of age on the bodyweight growth might overwhelm the impact of gender. Further investigations on the age influence are warranted. A number of factors contribute to the development of osteoporosis. With regard to disuse induced osteoporosis, the leading causal factor is the lack of mechanical loading. However, sex steroid also influences the responses of skeleton system to mechanical loading. It has been reported that testosterone could effectively prevent the bone loss in male mice receiving a unilateral botulinum toxin injection and orchidectomy [21]. In the current study, we observed a gender difference of the responses to mechanical unloading. The consequences after both hindlimb suspension and ovariectomization, as well as after estrogen supplementation warrant further investigation. More indices related to hormone level will be included in the future study to elucidate the correlation between estrogen and androgen to hindlimb unloading.

The impact of HLS on BMD was much greater for male rats than for female rats. As reported, the BMD value is only a surrogate measurement of bone strength and the risk of fracture would be underestimated if diagnosis of osteoporosis is made only on BMD [22]. Therefore, more indices are necessary for evaluation. By comparing the micro-architecture properties of trabecular femur between both genders, hindlimb suspension generated greater decrease in BV/TV, Tb.Th, Tb.N, and Conn. D for male rats. The enhancement in Tb.Sp was also greater in male rats. A similar gender difference in the effect of hindlimb unloading on bone architecture were also reported [23]. Osteoporosis is caused by the imbalance between bone formation and bone resorption. The anti-osteoporosis effect of ALE relies on its significant inhibition effect on bone resorption. The direct effect could be reflected by the micro-architecture via Micro-CT assay, demonstrated by the increased morphologic parameters, such as bone volume density (Figure 4A), trabecular thickness (Figure 4B), trabecular number (Figure 4C), as well as connectivity density (Figure 4E). As for the anti-osteoporosis mechanisms of RSE, in vitro studies suggested that baicalin (the major component in RSE) could enhance the osteogenic differentiation of rBMSC and osteoblast, which also induced the mere increase in bone formation with increased morphologic parameters [12]. However, it needs further investigation if RSE has an inhibition effect on bone resorption. For bone turnover markers in serum, BAP level was comparable in various treatment groups for female rats, whereas a significant increase was observed after hindlimb suspension for male rats.

Herbal medicines have been applied in the prevention and treatment of bone related diseases over a long history in China. A number of herbal medicines in the form of traditional formulas, single herb extract, or bioactive components have shown significant anti-osteoporosis effect [10]. Resveratrol, a natural compound abstracted from grape and Polygonumcuspidatum Sieb. et Zucc., showed significant preventive effect against bone loss induced by hindlimb immobilization in male Wistar rats and Fischer 344× Brown Norway rats at the dose of 400 and 12.5 mg/kg/day, respectively [24,25]. *Radix Scutellariae* is a traditional medicinal herb with strong pharmacological effects such as anti-inflammation and anti-oxidation [11,26]. In the present study, *Radix Scutellariae* extract (RSE), at a dose of 50 mg/body weight/day for 42 continuous days, effectively prevented and treated disuse induced osteoporosis in hindlimb suspended female rats, with increased BMD, improved micro-architectural properties of femoral trabecula, enhanced biomechanical strength, and decelerated bone remodeling induced by HLS treatment. For the establishment of administration doses, it is difficult to calculate the dose for rats from clinical dose since pharmaceutical excipients were added in the formulation clinically. Based on our previous study, Sprague-Dawley rats could bear the dose of RSE (200 mg/kg, t.i.d.) for five days [27]. Considering rats underwent a tail operation, the dose of 100 mg/kg/day was tried at first in a pilot study. Nevertheless, gastrointestinal symptoms (mainly diarrhea) were observed and several rats could not survive after administration for seven days. Therefore, a lower dose (50 mg/kg/day) was employed in the current study. During the experiment, no obvious toxicity was observed and the fur conditions and food intake of rats administrated with RSE were better than those of HLS rats without any drug administration.

As the precursor cell of osteoblast, rat bone marrow derived stem cells demonstrated reduced osteogenic differentiation ability after hind limb suspension, which might contribute to the occurrence of disuse-induced osteoporosis [28,29]. Our previous studies have demonstrated that baicalin is the most abundant bioactive flavones in *Radix Scutellariae* and it is the major flavonoid in blood circulation after oral administration of herbal formula containing RSE to Sprague-Dawley rats [30,31]. Besides, baicalin could promote osteoblast differentiation via Wnt/β-catenin signaling pathway in vitro, and its aglycone form baicalein showed strong inhibitory effect on osteoclast differentiation [12,13]. Therefore, subsequent in vitro studies were carried out to investigate if baicalin could enhance the osteogenic differentiation of rBMSC.

## 4. Materials and Methods

### 4.1. Chemicals and Reagents

*Radix Scutellariae* extract (RSE) containing 55.104 ± 1.759 of baicalin, 1.871 ± 0.009 of wogonoside, 4.037 ± 0.011 of oroxylin A-7-*O*-glucuronide, 1.431 ± 0.046 of baicalein, 0.382 ± 0.008 of wogonin and 0.124 ± 0.002 of oroxylin A (mg/100 mg), as well as baicalin with purity of 99% was purchased from Shanghai U-Sea Biotech Co., Ltd. (Shanghai, China). The bioactive components were quantified following our previous HPLC/UV method [30]. Alendronate sodium (ALE) was supplied by Sigma-Aldrich (Milwaukee, WI, USA). DMEM/F12 culture medium, fetal bovine serum, penicillin-streptomycin, and 0.25% Trypsin-EDTA were products of Hyclone. Sodium β-glycerophosphate (β-GP), dexamethasone (DEX), ascorbic acid (ASC), Alizarin Red, DMSO, and 3-(4,5-dimethylthiazol-2-yl)-2,5-diphenyltetrazolium bromide (MTT) were purchased from Sigma. Distilled and deionized water was used throughout the experiment.

### 4.2. Animals and Treatments

Female Sprague-Dawley rats (SIPPR-BK Experimental Animal Ltd., Shanghai, China) with age of 8 weeks were purchased from the Laboratory Animal Center of Xi’an Jiaotong University (School of Medicine, Xi’an, China). The rats were housed in an air-conditioned room (24 °C) under a 12/12 h light/dark cycle. They were fed with standard rodent chow containing 0.9% calcium and 0.7% phosphate and had free access to water. The experiments complied with the Guidelines for Animal Experimentation of Xi’an Jiaotong University, the Guidelines on the Care and Use of Laboratory Animals issued by the Chinese Council on Animal Research, and the Guide for the Care and Use of Laboratory Animals published by the National Institutes of Health (NIH publication No. 85-23, revised 2011, New York, NY, USA), and were conducted after the approval by the Laboratory Animal Administration Committee of Xi’an Jiaotong University (Institutional Animal Care and Use Committee, IACUC; Permit Number: XYJZS-1207011).

After one week of acclimatization, rats were randomly divided into four groups: control group (CON, *n* = 6), hindlimb-suspended group without treatment (HLS, *n* = 6) and hindlimb-suspended group treated with ALE and RSE (HLS-ALE and HLS-RSE, *n* = 6 each group), respectively. ALE and RSE was administrated to rats by intragastric gavage at a dose of 2 and 50 mg/kg/day, respectively. Rats in the CON and HLS groups received equal volumes of distilled water. Dosing lasted for 6 weeks and rats in the HLS, HLS-ALE, and HLS-RSE groups were treated by hindlimb-suspension from the third week following the procedures reported by Morey-Holton with modification [7].

At the end of experiment, rats were sacrificed and blood was collected by cardiac puncture. Blood was centrifuged at 2000 rpm at 4 °C for 20 min and serum samples were stored at −80 °C for biochemistry evaluation. Femurs were dissected and stored in saline at −20 °C for microCT assay and bone biomechanical tests.

### 4.3. Bone Mineral Density (BMD) Analysis

BMD at distal femoral metaphysic and proximal tibial metaphysic were measured by dual-energy X-ray absorptiometry (DXA) assay (Lunar Prodigy Advance DXA, GE healthcare, Madison, WI, USA) [32]. Data was calculated automatically by purpose-designed software (enCORE^TM^ 2006, GE Healthcare, Madison, WI, USA). Before measurement, rats were anesthetized by intraperitoneal injection with chloral hydrate at the dose of 300 mg/kg and fixed at repeatable positioning. Total BMD was measured including the cross-sectional area of both cortical and trabecular bones.

### 4.4. Micro-Computed Tomography (Micro-CT) Analysis

The micro-architecture of the left distal femur was analyzed by a desktop eXplore Locus SP Pre-Clinical Specimen microCT (GE Healthcare, Madison, WI, USA) with a little modification from the method reported by Qi et al. [20]. Femurs were aligned perpendicularly and scanned with a resolution of 12 μm voxel. Three-dimensional images were acquired from the microCT Evaluation Program. Trabecular bone was separated from cortical bone by free drawing regions of interests with the MicroView program (Version 2.1.2, GE healthcare, Madison, WI, USA) and a multiple Intel processor-based microCT workstation provided with the scanner. The volume of interest, 2 mm below the metaphyseal line, was chosen within 100 continuous slices. Morphologic measurements were performed and corresponding parameters were obtained including bone volume density (BV/TV), trabecular thickness (Tb.Th), trabecular number (Tb.N), trabecular separation (Tb.Sp), and connectivity density (Conn. D). Cortical thickness (Cr.Th) of the femur was also analyzed and compared among various groups.

### 4.5. Biomechanical Test

The three-point bending test was employed to determine the bone strength (MTS, Eden, Prairie, MN, USA) based on the method by Qi et al. [20]. Briefly, femurs were put on two supports spaced 20 mm apart and load was applied to the middle of the bone at a deformation rate of 2 mm/min until fracture occurred. The force and displacement data were recorded. The inner and outer width as well as the inner and outer height of the femur at the break point was also determined. A load-deformation curve was plotted when a load is applied to the femoral midshaft. The femur displacement (*d*) increases linearly with applied load (*F*). The maximum slope of the curve (Δ*F*/Δ*d*) is titled stiffness which represents the structural rigidity of the bone. When the maximum load is applied, bending further increases until a fracture occurs. Based on the initial linear part of the load deformation curve, Young’s modulus (maximum slope of the stress-strain curve, *E*) and maximum stress (*F*_max_/cross-sectional area, *σ*_max_) were calculated as the Equations (1) and (2) below:

*E* = (*FL*^3^)/(*d*48*I*) (1)

*σ*_max_ = (*FLD*)/(8*I*) (2)

where *F* is the maximum load (ultimate strength, *F*_max_), *L* is the distance between supporting points, *d* is the displacement, *I* is the moment of inertia of the cross-section in relation to the horizontal axis (Electronic supplementary material, ESM), and *D* is the outer height of femur.

### 4.6. Measurements of Serum Bone Turnover Markers

Serum calcium (Ca), phosphorus (P), Bone alkaline phosphatase (BAP), and titrate resistant acid phosphatase (TRACP) were quantified based on the method by Qi et al., with modification [20]. Ca and P were quantified by an automatic biochemical analyzer (CobasIntergra 400 plus, Roche Diagnostics, Basel, Switzerland) using the original kits from Roche Diagnostics (Indianapolis, IN, USA). BAP and TRACP in serum were quantified by ELISA kit (Beijing Sino-Uk Institute of Biological Technology, Beijing, China).

### 4.7. Cell Culture

Female Sprague-Dawley rats with the age of 8 weeks were provided by Laboratory Animal Center of Xi’an Jiaotong University (School of Medicine, Xi’an, China). Rats were decapitated and the tibia, as well as the femur were isolated [28]. Epiphyseal ends were cut and the marrow cavity was flushed with DMEM/F12. The eluent was collected and placed in still for 5 min. The supernatant was collected and centrifuged for 5 min at 1000 rpm. Rat bone marrow derived mesenchymal stem cells (rBMSC) were resuspended and cultured in DMEM/F12 supplemented with 10% fetal bovine serum, 100 U/mL of penicillin and 100 μg/mL of streptomycin in a humidified CO_2_ (5%) incubator at 37 °C. Differentiation was induced by the addition of ascorbic acid at 0.284 mM, sodium β-glycerophosphate at 10 mM, and dexamethasone at 2 μM.

### 4.8. Alkaline Phosphatase Assay

rBMSC was seeded to a 96-well plate at a density of 2 × 10^3^ cells/well. Cultured for 24 h, the medium was replaced by DMEM/F12 containing ASC, β-GP, and DEX in the presence or absence of baicalin at 0.1, 0.5, 1, 5, 10, and 50 μM. After being cultured for 3, 5, and 7 days, the medium was discarded and the plate was rinsed with PBS twice. The ALP activity was quantified by measuring the absorbance of generated yellow p-nitrophenol at 405 nm by using an alkaline phosphatase assay kit (Nanjing Jiancheng Bioengineering Institute, Nanjing, China) [28].

### 4.9. Mineralization Assay

rBMSC was seeded to a 24-well plate at a density of 2 × 10^5^ cells/well. Cultured for 24 h, the medium was replaced by DMEM/F12 containing ASC, β-GP, and DEX in the presence or absence of baicalin at 1, 10, and 50 μM. The formation of mineralized nodules were stained and analyzed according to the method by Ngueguim et al. [33]. After being cultured for 12 days, the medium was removed. Rinsed with PBS twice, cells were fixed with 10% of formaldehyde and stained by the addition of Alizarin Red at 40 mM. The area, number, and intensity of formed mineralized nodules were analyzed by Image-Pro Plus 6.0 (Media Cybernetics, Rockville, MD, USA).

### 4.10. Statistical Analysis

Data are presented as mean ± SD. All the statistical analysis was performed with the Statistical Package of Social Science (SPSS, Version 16.0, Chicago, IL, USA). Comparisons of group variance were performed by one-way ANOVA and Tukey’s *post hoc* test was performed for multiple comparisons. Statistical difference was set at *p* < 0.05.

## 5. Conclusions

To sum up, a hindlimb suspended rat model has been established. Mechanical unloading had a stronger impact on bone strength and micro-architecture in male Sprague-Dawley rats than the female rats. The extract of *Radix Scutellariae* demonstrated significant anti-osteoporotic capacity in vivo. The deteriorated condition of femoral trabecula induced by hindlimb suspension was effectively suppressed. Bone mineral density and bone biomechanical strength were significantly enhanced, indicating the potential application of *Radix Scutellariae* extract as an alternative supplement to prevent and treat disuse-induced osteoporosis.

## Figures and Tables

**Table 1:** Effect of RSE treatment on bone biomechanical parameters in rat femoral diaphysis for male and female rats.

Parameters	CON	HLS	HLS-ALE	HLS-RSE
Maximum stress (MPa)	111.24 ± 13.05	89.55 ± 5.28 *	109.84 ± 16.46 ^#^	116.37 ± 8.85 ^##^
Young’s modulus (MPa)	3576.52 ± 237.04	2724.98 ± 128.70 *	3415.29 ± 556.66 ^#^	3671.41 ± 314.43 ^#^
Maximum load (N)	77.02 ± 9.04	61.77 ± 3.70 **	80.05 ± 9.38 ^###^	80.57 ± 6.13 ^###^
Stiffness (N/mm)	198.96 ± 18.64	120.72 ± 11.50 ***	210.01 ± 26.34 ^###^	196.26 ± 13.39 ^###^

CON: control group; HLS: hindlimb suspended group; HLS-ALE: group administrated with alendronate (2 mg/kg/day); HLS-RSE: group administrated with *Radix Scutellariae* extract (50 mg/kg/day). Values are presented by means ± SD, *n* = 6; * versus CON group: * *p* < 0.05, ** *p* < 0.01, *** *p* < 0.001; ^#^ versus HLS group: ^#^ *p* < 0.05, ^##^ *p* < 0.01, ^###^ *p* < 0.001.

**Table 2:** Bone turnover markers in rat serum within various treatment groups (*n* = 6).

Parameters	CON	HLS	HLS-ALE	HLS-RSE
Ca (mM)	2.46 ± 0.10	2.41 ± 0.16	2.34 ± 0.123	2.37 ± 0.06
P (mM)	1.82 ± 0.08	2.08 ± 0.05	1.80 ± 0.21	2.04 ± 0.11
BAP (μg/L)	55.86 ± 1.55	55.03 ± 2.56	57.41 ± 1.25	56.29 ± 5.53
TRACP (pg/L)	2055.53 ± 172.20	2560.55 ± 114.58	2178.42 ± 287.16	2221.74 ± 203.29
		***	^#^	^#^

Ca: Calcium; P: phosphorus; BAP: bone alkaline phosphatase; TRACP: tartrate resistant acid phosphatase. CON: control group; HLS: hindlimb suspended group; HLS-ALE: group administrated with alendronate (2 mg/kg/day); HLS-RSE: group administrated with *Radix Scutellariae* extract (50 mg/kg/day). Values are presented by means ± SD, *n* = 6; * versus CON group: *** *p* < 0.001; ^#^ versus HLS group: ^#^ *p* < 0.05.

**Table 3:** Intensity scanning of mineralized nodule formation 12 days after osteogenic induction (*n* = 4).

Group	Area (mm^2^/Well)	Number (Mineralized Nodule/Well)	Intensity
CON	30.34 ± 6.53	702.78 ± 49.50	21955.34 ± 2397.71
1 μmol·L^−1^	54.55 ± 6.23 **	973.64 ± 74.70 **	71181.98 ± 5769.01 ***
10 μmol·L^−1^	68.14 ± 4.93 ***	1218.64 ± 147.13 **	90613.62 ± 6423.42 ***
50 μmol·L^−1^	64.03 ± 5.41 **	1157.68 ± 85.70 ***	74511.73 ± 5773.22 ***

** *p* < 0.01; *** *p* < 0.001 vs. CON.
